# Inhibitory Activity of Quercetin 3-*O*-Arabinofuranoside and 2-Oxopomolic Acid Derived from *Malus domestica* on Soluble Epoxide Hydrolase

**DOI:** 10.3390/molecules25184352

**Published:** 2020-09-22

**Authors:** In Sook Cho, Jang Hoon Kim, Yunjia Lin, Xiang Dong Su, Jong Seong Kang, Seo Young Yang, Young Ho Kim

**Affiliations:** 1 Department of Horticultural and Crop Environment, National Institute of Horticultural and Herbal Science, RDA, Wanju 55365, Korea; tuat@korea.kr; 2 Department of Herbal Crop Research, National Institute of Horticultural and Herbal Science, RDA, Eumseong 27709, Korea; oasis5325@gmail.com; 3 College of Pharmacy, Chungnam National University, Daejeon 34134, Korea; linyunjia1995@163.com (Y.L.); kangjss@cnu.ac.kr (J.S.K.); 4 School of Pharmaceutical Sciences (Shenzhen), Sun Yat-sen University, Guangzhou 510275, China; suxd7@mail.sysu.edu.cn

**Keywords:** *Malus domestica*, soluble epoxide hydrolase, mixed inhibitor, noncompetitive inhibitor, binding pocket

## Abstract

Flavonoids and triterpenoids were revealed to be the potential inhibitors on soluble epoxide hydrolase (sEH). The aim of this study is to reveal sEH inhibitors from Fuji apples. A flavonoid and three triterpenoids derived from the fruit of *Malus domestica* were identified as quercetin-3-*O*-arabinoside (**1**), ursolic acid (**2**), corosolic acid (**3**), and 2-oxopomolic acid (**4**). They had half-maximal inhibitory concentration of the inhibitors (IC_50_) values of 39.3 ± 3.4, 84.5 ± 9.5, 51.3 ± 4.9, and 11.4 ± 2.7 μM, respectively, on sEH. The inhibitors bound to allosteric sites of enzymes in mixed (**1**) and noncompetitive modes (**2**–**4**). Molecular simulations were carried out for inhibitors **1** and **4** to calculate the binding force of ligands to receptors. The inhibitors bound to the left (**1**) and right (**4**) pockets next to the enzyme’s active site. Based on analyses of their molecular docking and dynamics, it was shown that inhibitors **1** and **4** can stably bind sEH at 1 bar and 300 K. Finally, inhibitors **1** and **4** are promising candidates for further studies using cell-based assays and in vivo cardiovascular tests.

## 1. Introduction

Arachidonic acid is converted to epoxyeicosatrienoic acids (EETs) by cytochrome P450 epoxygenase [1]. EETs exist as four regioisomeric metabolites: 5,6-, 8,9-, 11,12-, and 14,15-EET [1]. Soluble epoxide hydrolase (sEH, E.C.3.3.2.10) biosynthesizes dihydroxyeicosatrienic acids (DHETs) from EETs by hydrolyzing their epoxide rings into a diol [2]. EETs were first revealed to be endothelium-derived hyperpolarizing factors in 1996 and have since been found to have various biological activities, including anti-inflammatory, antihypertensive, kidney-protective, and cardiac-protective effects [3]. 11,12-EET was reported to inhibit nuclear factor kappa B signaling pathways and the cytokine-induced expression of cellular adhesion molecules [4]. 11,12-/14,15-EETs stimulate the large-conductance Ca^2+^-activated K^+^ channel and expand blood vessels [3]. Thus, sEH, which is encoded in the EPHX2 gene located at chromosomal region 8p21-p12, has gained attention as a therapeutic target for the treatment of cardiovascular disease [5]. 12-(3-adamantan -1-yl-ureido)dodecanoic acid (AUDA) is an effective sEH inhibitor [6]. Owing to its low solubility, AUDA is dissolved in dimethyl sulfoxide for in vitro experiments and formulated with large amounts of 2-hydroxylpropyl β-cyclodextrin for in vivo use [3]. Therefore, the aim of this study is to find sEH inhibitors that have higher solubility than that of AUDA.

The apple is one of the most widely consumed fruits worldwide [7]. The Fuji apple (*Malus domestica* Brokh.), which originated in Japan in 1962 [8], is the most consumed apple variety in Korea [9]. This fruit has been reported to contain phenolic compounds such as caffeoylquinic acid, epicatechin, and procyanidins, which provide health benefits such as decreased risk of cardiovascular disease and cancer [10]. Recently, triterpenoids from *Cimicifuga dahurica* Maxim. [11], flavonoids from *Tetrastigma hemsleyanum* [12], and flavonoid glycosides [13] have been reported as noncompetitive agents blocking the catalytic activity of sEH [12,13]. In the present study, flavonoid and triterpenoid compounds were isolated from apples, and their structures were identified using spectroscopic methods. Isolated compounds were evaluated to assess their inhibitory activity against sEH and beneficial effects for cardiovascular disease.

## 2. Results and Discussion

### 2.1. Isolation, Identification, and Enzyme Assay

**Figure 1:**
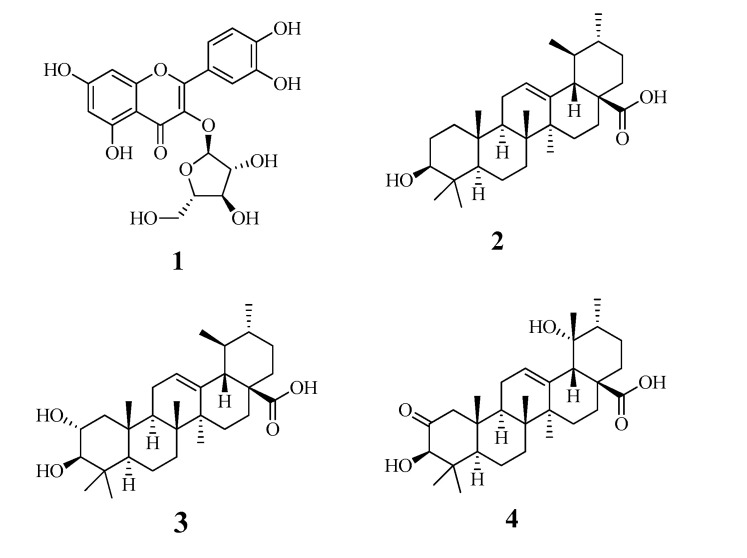
The structures of compounds **1**–**4** isolated from the fruits of *Malus domestica*.

In recent studies, we revealed that flavonoids and triterpenoids have inhibitory activity against sEH [11,12,13]. A flavonoid glycoside (**1**) and triterpenoids (**2**–**4**) were purified from the fruit of *M. domestica*. Their chemical structures were identified as quercetin 3-*O*-arabinofuranoside (**1**) (Figure S1) [14], ursolic acid (**2**) (Figure S2) [15], corosolic acid (**3**) (Figure S3) [16], and 2-oxopomolic acid (**4**) (Figure S4) [17] based on nuclear magnetic resonance (NMR) analysis and comparisons with the reported data (Figure 1).

**Figure 2:**
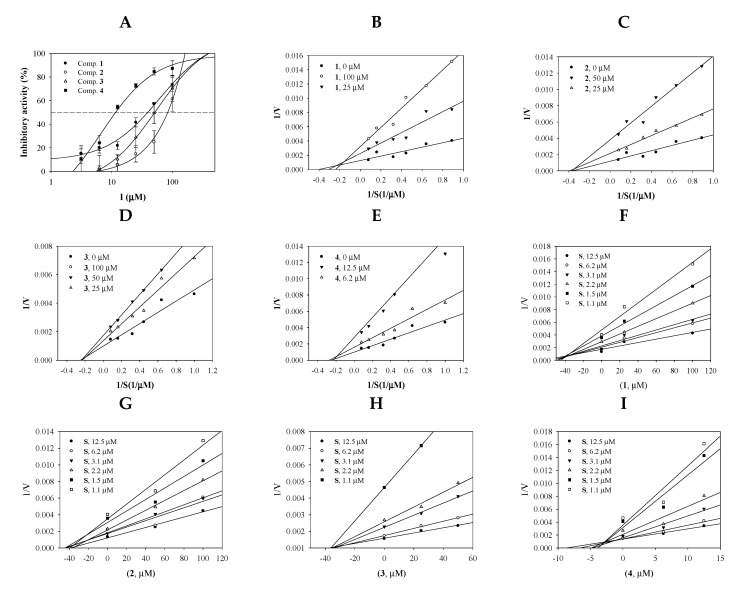
Inhibitory activities of the inhibitors **1**–**4** at a variety of concentrations toward soluble epoxide hydrolase (sEH) (**A**). Lineweaver-Burk plots (**B**–**F**) and Dixon plots (**G**–**I**) for the inhibition of sEH by compounds **1**–**4**.

Purified compounds **1**–**4** (Figure S5) were evaluated to assess their sEH-inhibitory activity in vitro. The inhibitory activity of each concentration of compound was calculated based on the difference in enzyme products with and without the presence of the compound using Equation (1). Figure 2A shows that the compounds had sEH inhibition activity in a dose-dependent manner at 3.1–100 μM concentrations. To calculate the half-maximal inhibitory concentration of the inhibitors (IC_50_), inhibition rate values were fitted using Equation (2). Inhibitors **1**–**4** were found to have IC_50_ values of 39.3 ± 3.4, 84.5 ± 9.5, 51.3 ± 4.9, and 11.4 ± 2.7 μM, respectively (Table 1). AUDA was used as the positive control (1.5 ± 1.2 nM).

The enzyme kinetic study was performed to find the binding position of the inhibitor into sEH. The initial velocity (*v*_0_) for the three different concentrations of inhibitors was calculated relative to the substrate concentrations (1.1, 1.5, 2.2, 3.1, 6.2, and 12.5 μM). The inverse of these concentrations and *v*_0_ values were used to construct Lineweaver-Burk plots. As indicated in Figure 2B–E, flavonoid **1** and triterpenoids **2**–**4** exhibited mixed inhibitions (different −1/*K*m and 1/*V*max values) and noncompetitive inhibitions (a −1/*K*m value and different 1/*V*max values) (Table 1).

As presented in Figure 2F–I and Table 1, the inhibition constant (*k*_i_) values were calculated by Dixon plots. Inhibitors 1–4 were calculated to be *k*_i_ values ranging from 2.9 ± 0.8 μM to 39.5 ± 1.0 and 38 μM. Among them, inhibitor **4** had the strongest *k*_i_ value of 2.9 ± 0.8 μM.

### 2.2. Molecular Docking

**Figure 3:**
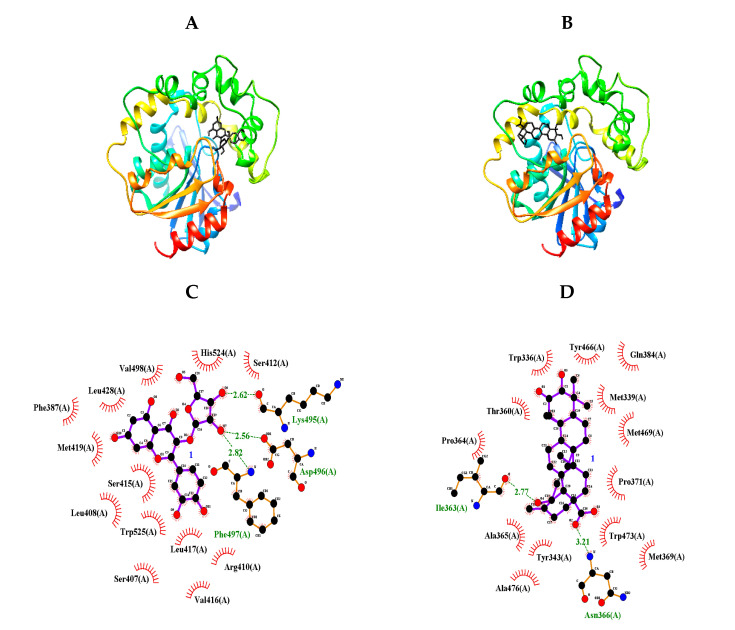
Predicted docking poses between compounds **1** and **4** and sEH (**A**,**B**). Hydrogen bonds of inhibitors **1** and **4** of the catalytic site, respectively (**C**,**D**).

In 1894, the “lock-and-key” model was first suggested by Emil Fisher to describe the interactions of enzymes (locks) with substrates (keys) [18]. Molecular docking is a virtual technology that simulates these interactions, and such computer technologies have been used for drug development by Merck Inc. since the 1980s [19]. We carried out molecular docking analyses using the Autodock 4.2 program to gain insight into the inhibitor-sEH interactions. Based on the enzyme kinetic results, blind docking was performed to identify an allosteric site to which inhibitors **1** and **4** with IC_50_ values below 50 μM could bind. Two compounds had the highest-scoring poses, with binding energies of −9.03 and −10.18 kcal/mol, respectively (Figure 3A,B and Table 2). As shown in Figure 3C and Table 2, flavonoid glycoside (**1**) had three hydrogen bonds between its sugar group and Lys495 (at a 2.62-Å distance), Asp496 (at a 2.56-Å distance), and Phe497 (at a 2.82-Å distance). Triterpenoid (**4**) formed two hydrogen bonds: between its carboxyl group and Asn366 at a distance of 3.21 Å and between its hydroxyl and ILe363 at a distance of 2.77 Å (Figure 3D and Table 2). This hydroxyl unit is a functional group only in compound **4** among triterpenoids. It can be confirmed that the sEH inhibitory activity increases as this functional group binds to the enzyme. Through a molecular docking study, the allosteric sites were identified as the left (**1**) and right (**4**) pockets next to the catalytic site, respectively.

### 2.3. Molecular Dynamics

**Figure 4:**
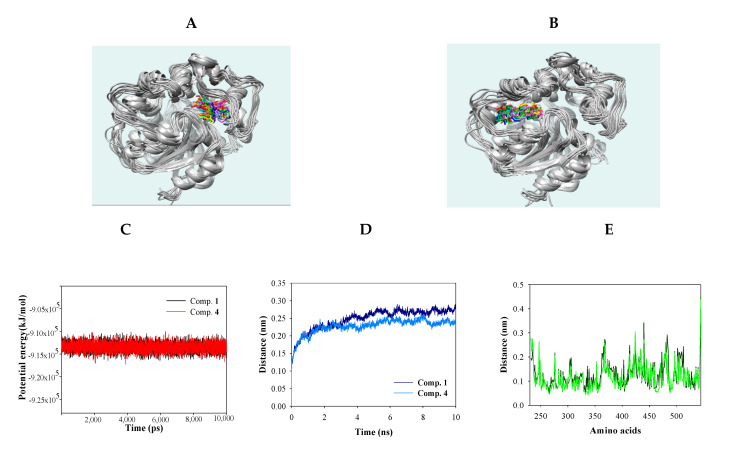

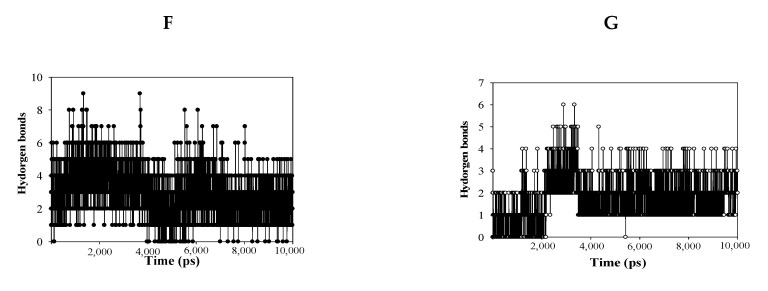
Superpositions of sEH with compounds **1** and **4** for simulation trajectory (0 ns: red, 1: orange, 2: yellow, 3: green, 4: forest green, 5: cyan, 6: blue, 7: cornflower blue, 8: purple, 9: hot pink, and 10: magenta) (**A**,**B**). The potential energy (**C**), root mean square deviation (RMSD) (**D**), root mean square fluctuation (RMSF) (**E**), and hydrogen bonds (**F**,**G**) of the simulation calculated with **1** and **4** during 10 ns.

Molecular dynamics (MD) simulation was used to obtain computational information on the bovine pancreatic trypsin inhibitor under vacuum conditions by McCammon in 1977 [19]. MD is a state-of-the-art computational technique for investigating interactions between ligands and receptors [19]. The “induced fit” model of enzyme-inhibitor binding was proposed by Koshland, as inhibitors can induce enzyme conformation changes during binding [19]. NMR and X-ray crystallography have provided limited insight into the static binding pose of ligands and enzymes [20]. In the present study, MD simulation using the Gromacs 4.6 program was carried out to assess the interactions between the inhibitors (**1** and **4**) and sEH. As shown in Figure 4A,B, inhibitors **1** and **4** bound with sEH, reaching a steady state with ~9.1 × 155 kJ/mol potential energy (Figure 4C). The protein root mean square deviation (RMSD) values of compounds **1** and **4** were ~2.8 and ~2.5 Å, respectively (Figure 4D). Amino acids that bonded with the two compounds exhibited root mean square fluctuation (RMSF) values within ~3.5 Å (Figure 4E). Inhibitors **1** and **4** maintained mainly 1–5 and 1–3 hydrogen bonds with sEH residues for 10 ns (Figure 4F,G). Hydrogen bonds in sEH-inhibitor complexes were analyzed at 1-ns intervals during their trajectory (Table 3). As a result, inhibitor **1** consistently formed hydrogen bonds with four residues: Ser415, Val416, Met419, and Lys495. Inhibitor **4** was found to form hydrogen bonds with Tyr343 and Ser374 continuously. These results were different from the ligand-receptor hydrogen bond in molecular docking. These facts suggest that the two inhibitors affect the enzymatic structure. Accordingly, inhibitors **1** and **4** were assigned to the “induced fit” in “lock-and-key” models. In silico, it was confirmed that the hydrogen bonds of the inhibitors were different in stationary (docking) and fluid (dynamic) enzymes. These results suggest that the two compounds bind to the enzyme by changing the enzyme structure as the “induced fit” theory.

**Figure 5:**
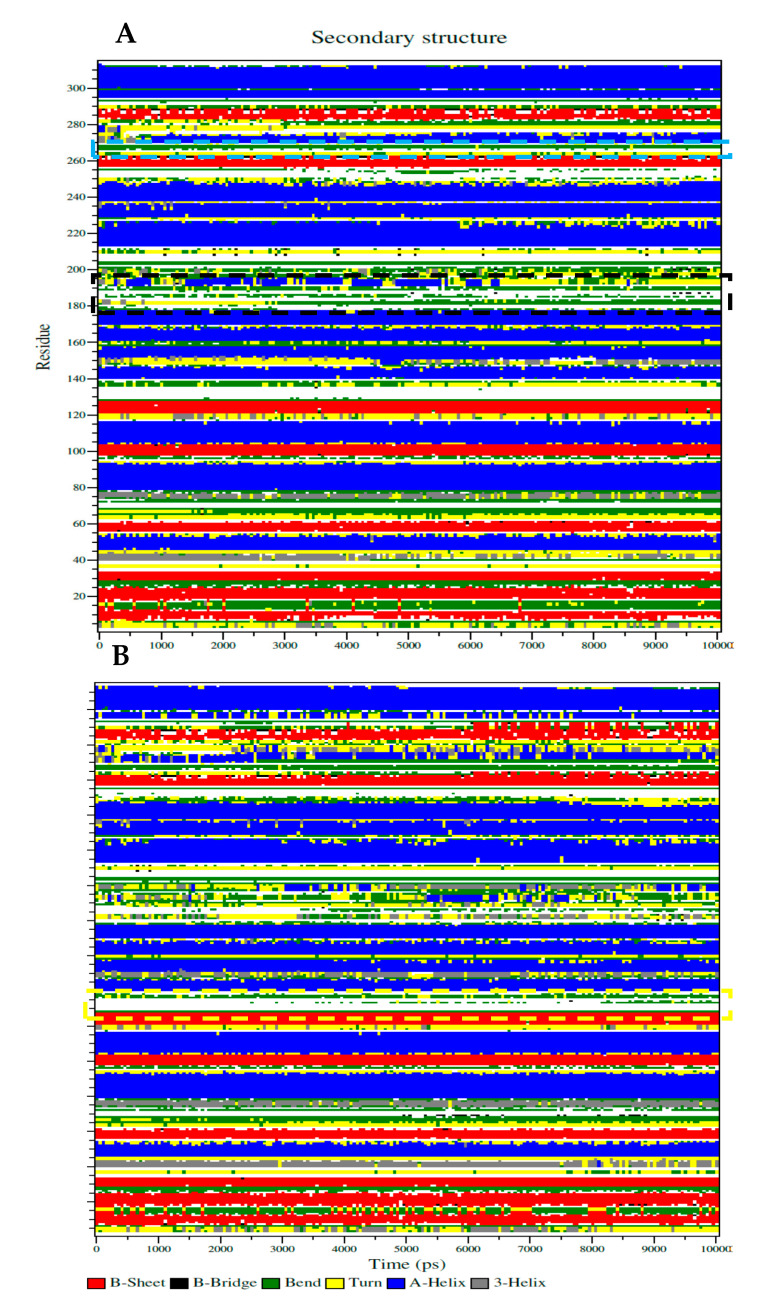
The Dictionary of Secondary Structure protein (DSSP) represented with secondary structure changes of sEH with inhibitor **1** (**A**) or **4** (**B**) (yellow square: Thr360-Pro371, black square: Ser415-Glu424, and blue square: Glu494-Pro501).

The sEH-inhibitor complexes that were analyzed by the Dictionary of Secondary Structure protein (DSSP) of **1** depended on the Ser415-His420, Lys495-Val500, and Cys522-Trp525 loops in the left pocket (Figure 5A), as well as **4** anchored to the Thr360-Pro371 loop when binding to the right pocket (Figure 5B). As shown in Figure 5A,B, this loop maintained a coil and band structure without any major structural changes. This finding confirms that the inhibitor is the correct size for the loop pocket (Figure 3A). The key amino acids-containing loop (Ser415, Val416, and Met419) in sEH was partially transformed into various secondary structures in the form of a bend, turn, and α-helix by compound **1**, whereas the Lys495 loop showed a fixed bend and turn structure (Figure 5A). As indicated in Figure 5B, the Thr360-Pro371 loop was retained as a fixed secondary structure despite binding to inhibitor **4**. These facts confirmed that, after binding of the sEH and inhibitors, the secondary structure of the loop containing key amino acids was not affected. 

## 3. Materials and Methods

### 3.1. General Experimental Procedures

Column chromatography were performed using silica gel (Kieselgel 60, 70–230, and 230–400 mesh, Merck, Darmstadt, Germany); Sephadex LH-20 (GE Healthcare, Uppsala, Sweden); and C-18 (ODS-A 12 nm S-150 and S-75 μm; YMC Co., Dinslaken, Germany) resins. Thin-layer chromatography (TLC) was performed using precoated silica gel 60 F254 and RP-18 F254S plates (both 0.25 mm, Merck). Spots in TLC were visualized by spraying with 10% aqueous H_2_SO_4_ solution, followed by heating at 300 °C dried air. Nuclear magnetic resonance (NMR) spectra were recorded using the JEOL ECA 500 spectrometer (^1^H, 500 MHz and ^13^C, 125 MHz) (Tokyo, Japan). AUDA (10007927), soluble epoxide hydrolase (10011669), and 3-phenyl-cyano(6-methoxy-2-naphthalenyl) methyl ester-2-oxiraneacetic acid (PHOME) (10009134) were purchased from Cayman (Cayman, Ann Arbor, MI, USA).

### 3.2. Plant Materials

The fruits of *M. domestica* were collected in October 2017 at Jansu-gun, Jeollabuk-do, Republic of Korea and were identified by Prof. Y.H. Kim in the College of Pharmacy, Chungnam National University, Daejeon, Republic of Korea. A voucher specimen (CNU 17003) was deposited at the Herbarium of the College of Pharmacy, Chungnam National University.

### 3.3. Extraction and Isolation

The flesh (peel and seeds removed) and peel of *M. domestica* were sliced and dried at room temperature for one week. The dried flesh (1.6 kg) and peel (260.0 g) were extracted three times with methanol by ultrasonic solvent extraction in an ultrasound bath at 35 ± 3 °C. After removal of solvents under reduced pressure, methanol extracts of the flesh (725.0 g) and peel (36.0 g) were obtained. Two methanol extracts were suspended in distilled water and successively partitioned with *n*-hexane, ethyl acetate, and *n*-BuOH to yield two *n*-hexane fractions (FH and PH), two ethyl-acetate fractions (FE and PE), and two *n*-BuOH fractions (FB and PH), respectively. The PE fraction (16.2 g) was subjected to silica gel column chromatography with a gradient of CH_2_Cl_2_-MeOH (30:1→1:1) to yield eight fractions (PE1–PE8). Compound **1** (20.0 mg) was isolated from the PE8 fraction using RP-18 silica gel column chromatography with MeOH-H_2_O (2:1→1:1) as the gradient elution solvent. The FE fraction (18.1 g) was chromatographed using vacuum liquid column chromatography and eluted with a CH_2_Cl_2_-MeOH (50:1→1:1) gradient system to yield six fractions (FE1–FE6). The FE2 fraction was chromatographed using RP-18 silica gel column chromatography and eluted with a MeOH-H_2_O (3:1) isocratic system to yield compound **4** (16.3 mg). The FE3 fraction was separated using RP-18 silica gel column chromatography with MeOH-H_2_O (3:1) to yield compound **2** (10.1 mg) and compound **3** (1.5 mg), respectively.

### 3.4. sEH Assay and Kinetic Analysis

The assays were carried out at 37 °C in 0.025-M bis-tris-HCL buffer, pH 7.0, containing 0.1% Bovine Serum Albumin B. PHOME (~2.6 μM) was used as a substrate. The final volume of tests was 200 μM in ~10 ng/mL sEH. The products were monitored at 330 nm of excitation and 465 nm emission for 40 min.

The inhibition rate was calculated according to the following equations:

Inhibition effect rate (%) = [(ΔC − ΔS)/ΔC] × 100(1)

where ΔC and ΔS represent the gap of control and inhibitor during reaction, respectively.

y = y_0_ + [(a × x)/(b + x)] (2)

where “y_0_” is the point on the axis of ordinates, “a” denotes the difference between the maximum and minimum values, and “b” refers to the “x” value at 50%.

### 3.5. Docking Study of sEH with Inhibitor

The 3D structure of the inhibitor was downloaded from the ChemSpider database and then minimized by MM2. The single bonds were designated as rotational bonds by using AutoDockTools. The 3D structure of sEH was downloaded from the research collaboratory for structural bioinformatics protein data bank coded with 3ANS ID. A chain of that with hydrogen atoms and Gasteiger charges was used for docking. The grid dimensions were set at 126 × 126 × 126 number of points (x × y × z). Docking simulation was performed to dock the inhibitor 25,000,000 times into this grid. The results of this study were represented as a diagram by using Chimera (San Francisco, CA, USA) and Ligplot (Cambridge, UK).

### 3.6. Molecular Dynamic Study

To simulate the molecular dynamics of sEH with the inhibitor, the Gromacs program (version 4.6.5) was used. The Itp and gro files of the inhibitor were created in the GlycoBioChem PRODRG2 server. sEH gro (Gromos96 43a1 force field) and topology files were encoded by gdb2gmx. The complex between the inhibitor and sEH was contained as cubes of water molecules measuring 8.5 × 8.5 × 8.5 containing six chloride ions. The energy minimization of that was lowered by 10 kJ/mol maximal force in the steepest descent method. A stable simulated complex was progressively simulated 300 K and at 1 bar for 100 ps, respectively. Finally, the stable product was subjected to molecular dynamics for 10 ns.

### 3.7. Data Analysis

Data were expressed as the means ± standard deviation (*n* = 3). All values were analyzed using SigmaPlot to determine the treatment variations.

## 4. Conclusions

A flavonoid and three triterpenoids from Fuji apples were indicated to have an inhibitory activity at 100 μM toward sEH. Among them, inhibitors **1** and **4** were calculated as IC_50_ values with 39.3 ± 3.4 and 11.4 ± 2.7 μM, respectively. These inhibitors were interacted into the position next to the catalytic site of the enzyme. Especially, the findings in the molecular simulation suggest that these two compounds bind to the enzyme by changing the enzyme structure as in the “induced fit” theory. These facts confirmed the possibility that quercetin-3-*O*-arabinoside (**1**) and 2-oxopomolic acid (**4**) contained in the fruits of *M. domestica* are natural sEH inhibitors in vitro and in silico. Inhibitors **1** and **4** are promising candidates for further studies using cell-based assays and in vivo cardiovascular tests.

## Figures and Tables

**Table 1:** The soluble epoxide hydrolase (sEH) inhibitory activities of compounds **1**–**4**.

	The Inhibitory Activity on sEH	
	IC_50_ Value ^a^ (μM)	Binding Mode, *k*_i_ (μM)
**1**	39.3 ± 3.4	Mixed (39.5 ± 1.0)
**2**	84.5 ± 9.5	Noncompetitive (38.5 ± 3.7)
**3**	51.3 ± 4.9	Noncompetitive (34.6 ± 1.6)
**4**	11.4 ± 2.7	Noncompetitive (2.9 ± 0.8)
AUDA ^b^	1.5 ± 1.2 (nM)	

^a^ All compounds were tested in a set of triplicated experiments. ^b^ Positive control. IC_50_: half-maximal inhibitory concentration of the inhibitors, AUDA: 12-(3-adamantan-1-yl-ureido)dodecanoic acid, and *k*_i_: inhibition constant.

**Table 2:** Interaction and binding energy of sEH with inhibitors **1** and **4**.

	Hydrogen Bonds (Å)	Binding Energy (kcal/mol)
**1**	Lys495 (2.62), Asp496 (2.56), Phe497 (2.82)	−9.03
**4**	Ile363 (2.77), Asn366 (3.21)	−10.18

**Table 3:** Hydrogen bond analysis of the inhibitor with sEH at 1-ns intervals for 10 ns.

Time (ns)	1	4
	Amino Acid (Å)	Amino Acid (Å)
**0**	Ser407(3.03), Ser415(3.27), Leu417(3.14), Phe497(2.82), Lys495(3.06), Val416(3.26)	Ile363(3.27)
**1**	Val416(3.10), Glu414(3.12), Ser418(2.46), Met419 (3.12), Lys495(3.10), Ser415(2.94), Phe497(3.09)	Tyr343(2.88)
**2**	Val416(2.81), Ala411(2.89), Lys495(3.12), Ser418(2.45), Met419(2.79)	Ile363(3.29), Tyr343(2.74)
**3**	Val416(2.93), Met419(3.01), Ser418(3.02), Ser415(2.92), Lys495(2.96)	Thr360(2.75), Trp473(2.90), Ser374(2.61)
**4**	Glu414(2.68,2.90), Val416(2.84), Met419(3.22), Ser415(2.95), Lys495(2.76)	Ser374(2.67)
**5**	Lys495(2.80), Phe497(3.09)	Ser374(2.73)
**6**	Val416(3.31), Met419(2.72), His420(2.89), Lys495(3.13)	Tyr343(2.61), Ser374(2.65)
**7**	Glu414(2.66,3.29), Val416(3.14), Met419(2.90)	Tyr343(3.35), Ser374(2.57)
**8**	Asp413(2.97), Val416(2.81), Met419(2.93)	Tyr343(3.18), Ser374(2.59)
**9**	Val416(2.85), Ser407(3.02), Met419(2.76), Ser415(2.93)	Ser374(2.79)
**10**	Val416(2.87), His524(3.02), Lys495(3.19), Ser415(3.33), Leu417(3.10), Met419(2.80)	Ser374(2.60), Thr360(2.79)

## Supplementary Materials

Figure S1: ^1^H/^13^C-NMR spectra of **1**. Figure S2: ^1^H/^13^C-NMR spectra of **2**. Figure S3: ^1^H/^13^C-NMR spectra of **3**. Figure S4: ^1^H/^13^C-NMR spectra of **4**. Figure S5: C-18 TLC results of compounds **1**–**4**.
