# Supplementary material for: Inhibitory Activity of Quercetin 3-O-Arabinofuranoside and 2-Oxopomolic Acid Derived from Malus domestica on Soluble Epoxide Hydrolase
Source: Molecules. 2020 Sep 22;25(18):4352. doi: 10.3390/molecules25184352 (PMC7576482; doi:10.3390/molecules25184352)
Supplement: Supplementary file 1 [file molecules-25-04352-s001.pdf]

molecules

Supplementary Materials

Inhibitory Activity of Quercetin 3-*O*-Arabinofuranoside and 2-Oxopomolic Acid Derived from *Malus domestica* on Soluble Epoxide Hydrolase

In Sook Cho [1,†], Jang Hoon Kim [2,†], Yunjia Lin [3], Xiang Dong Su [4], Jong Seong Kang [3], Seo Young Yang [3,*] and Young Ho Kim [3,*]

[1] Department of Horticultural and Crop Environment, National Institute of Horticultural and Herbal Science, RDA, Wanju 55365, Korea; tuat@korea.kr

[2] Department of Herbal Crop Research, National Institute of Horticultural and Herbal Science, RDA, Eumseong 27709, Korea; oasis5325@gmail.com

[3] College of Pharmacy, Chungnam National University, Daejeon 34134, Korea; linyunjia1995@163.com (Y.L.); kangjss@cnu.ac.kr (J.S.K.)

[4] School of Pharmaceutical Sciences (Shenzhen), Sun Yat-sen University, Guangzhou 510275, China; suxd7@mail.sysu.edu.cn

* Correspondence: syyang@cnu.ac.kr (S.Y.Y.); yhk@cnu.ac.kr (Y.H.K.). Tel.:+82-42-821-7321 (S.Y.Y.); Tel.:+82-42-821-5933 (Y.H.K.); Fax: +82-42-823-6566 (S.Y.Y. & Y.H.K.)

† These authors contributed equally to this work.

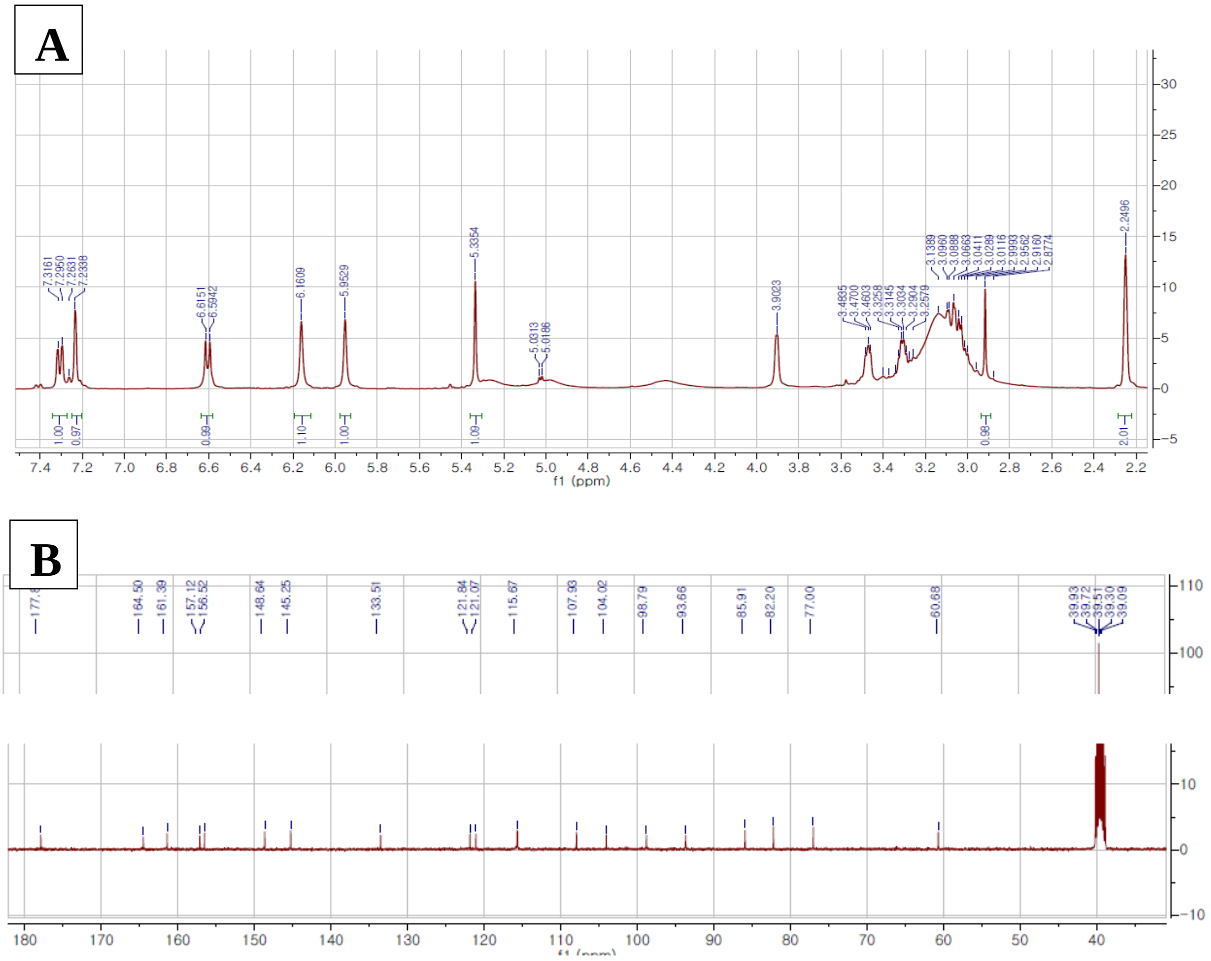

Figure S1. ^{1}H(A) /^{13}C(B)-NMR spectra of compound **1** (DMSO-d_6).

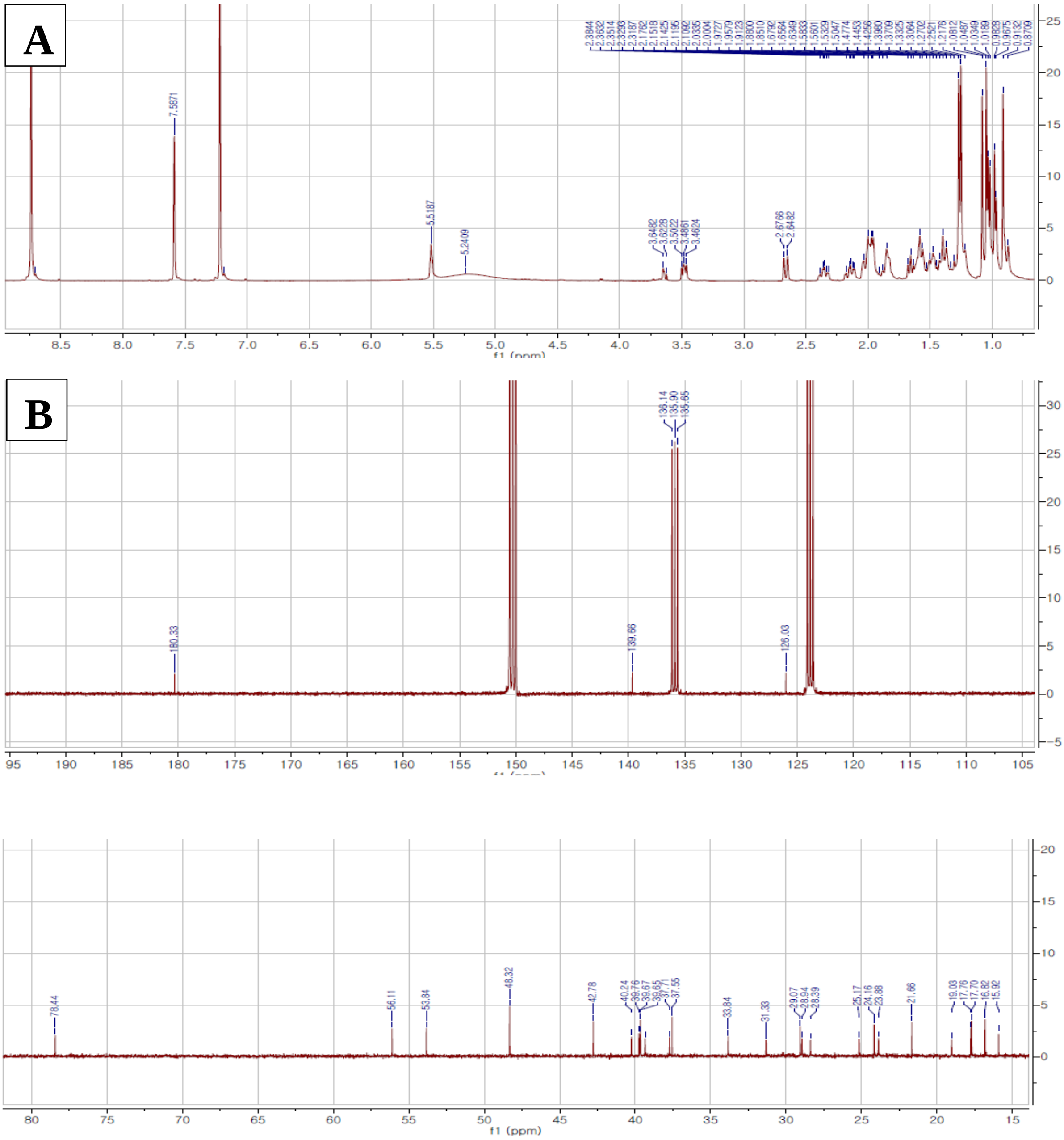

Figure S2. ^{1}H(A) /^{13}C(B)-NMR spectra of compound **2** (Pyridine-d_5).

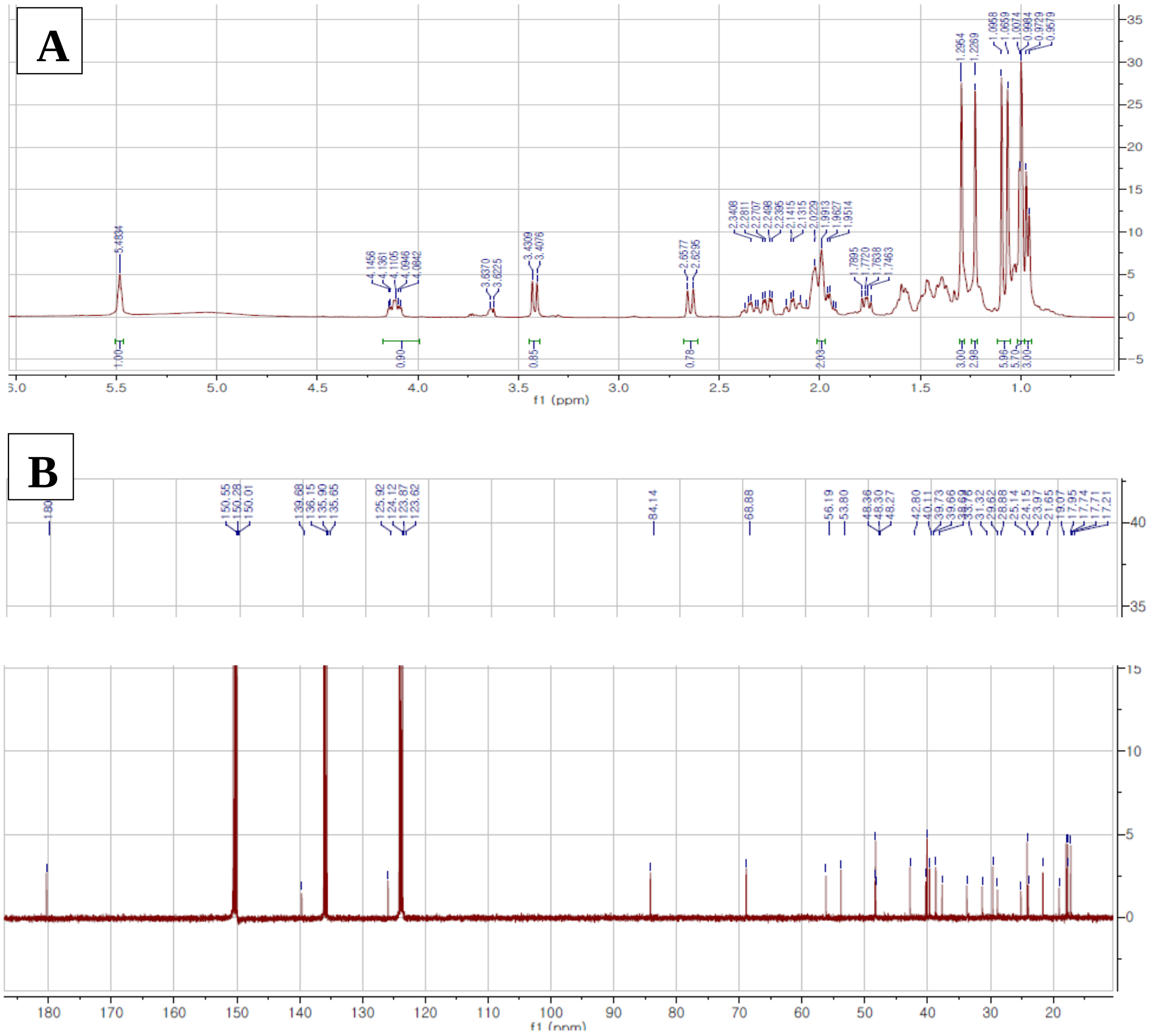

Figure S3. ^{1}H(A) and ^{13}C(B)-NMR spectra of compound **3** (Pyridine-*d*5).

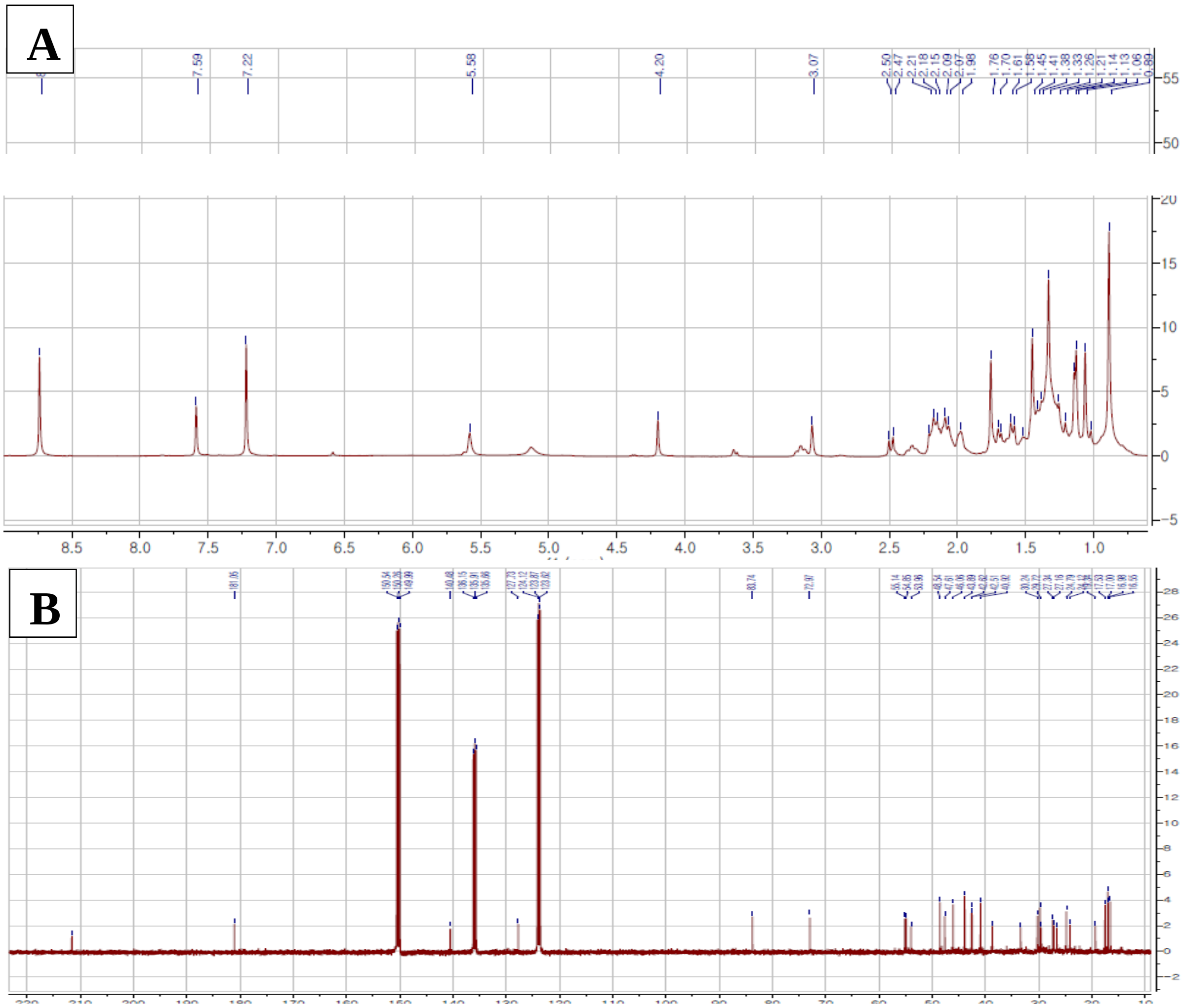

Figure S4. ^{1}H(A) and ^{13}C(B)-NMR spectra of compound **4** (Pyridine-d_5).

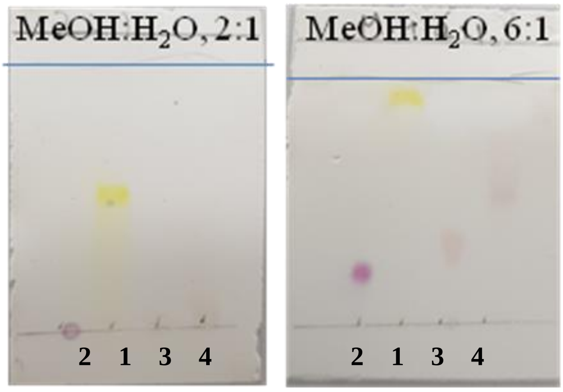

Figure S5. C-18 TLC results of compounds **1**-**4**.
